# Diurnal Microstructural Variations in Healthy Adult Brain Revealed by Diffusion Tensor Imaging

**DOI:** 10.1371/journal.pone.0084822

**Published:** 2014-01-06

**Authors:** Chunxiang Jiang, Lijuan Zhang, Chao Zou, Xiaojing Long, Xin Liu, Hairong Zheng, Weiqi Liao, Yanjun Diao

**Affiliations:** Paul C. Lauterbur Research Center for Biomedical Imaging, Shenzhen Institutes of Advanced Technology, Chinese Academy of Sciences, Shenzhen, China; Imperial College London, United Kingdom

## Abstract

Biorhythm is a fundamental property of human physiology. Changes in the extracellular space induced by cell swelling in response to the neural activity enable the in vivo characterization of cerebral microstructure by measuring the water diffusivity using diffusion tensor imaging (DTI). To study the diurnal microstructural alterations of human brain, fifteen right-handed healthy adult subjects were recruited for DTI studies in two repeated sessions (8∶30 AM and 8∶30 PM) within a 24-hour interval. Fractional anisotropy (FA), apparent diffusion coefficient (ADC), axial (λ_//_) and radial diffusivity (λ_⊥_) were compared pixel by pixel between the sessions for each subject. Significant increased morning measurements in FA, ADC, λ_//_ and λ_⊥_ were seen in a wide range of brain areas involving frontal, parietal, temporal and occipital lobes. Prominent evening dominant λ_⊥_ (18.58%) was detected in the right inferior temporal and ventral fusiform gyri. AM-PM variation of λ_⊥_ was substantially left side hemisphere dominant (p<0.05), while no hemispheric preference was observed for the same analysis for ADC (p = 0.77), λ_//_ (p = 0.08) or FA (p = 0.25). The percentage change of ADC, λ_//_, λ_⊥_, and FA were 1.59%, 2.15%, 1.20% and 2.84%, respectively, for brain areas without diurnal diffusivity contrast. Microstructural variations may function as the substrates of the phasic neural activities in correspondence to the environment adaptation in a light-dark cycle. This research provided a baseline for researches in neuroscience, sleep medicine, psychological and psychiatric disorders, and necessitates that diurnal effect should be taken into account in following up studies using diffusion tensor quantities.

## Introduction

Circadian rhythm is a fundamental property of human physiology at both cellular and organism levels. For example, body temperature rises in the morning and falls in the night, blood pressure and heart rate begin increasing and the adrenal cortex starts to secrete more cortisol before we wake up [1]–[3]. These cycles synchronize daily activities to the biological functions and enable better adaptation to cyclic changes in the environmental conditions for creatures.

Brain harbors essential systems of biological clocks that regulate human circadian physiology with known or unknown mechanisms [4]. Disruption of these rhythms can profoundly influence human health and induce metabolic disorders, insomnia, depression and cardiovascular diseases [5], [6]. However, the daily and seasonal cycles are not passive responses to the world around us, they are predominantly driven by endogenous neural mechanisms as part of the fundamental brain function [1]. Many investigations have confirmed that in mammals the primary circadian clock is located in the suprachiasmatic nuclei (SCN) of the hypothalamus, which in turn synchronizes peripheral oscillators throughout the brain and body [5], [7], [8]. The interactions among brainstem reticular activating system, thalamus pacemaker nucleus and cerebral cortex constantly modulate the metabolic and behavioral states of human body through diffusion of neuroactive substances in the intracellular and exatracellular space [9], [10]. The microscopic organization of structural barriers such as cell membrane, axons and macromolecules may channel the substances moving in a certain direction, which provides the physical bases for the noninvasive characterization the cerebral microstructure employing the diffusion properties of water molecule [11].

Diffusion tensor imaging (DTI) [12], [13], as an ideal tool in exploring brain physiology [14], [15] and pathology [16]–[21], is sensitive to the Brownian motion of water as it diffuses within tissues, thus providing exquisite microstructural details of tissue through measuring the diffusion quantities in vivo. The derived parameters including fractional anisotropy (FA), diffusivity and apparent diffusion coefficient (ADC) intrinsic to tissue cellularity have been employed to evaluate the cell size and microdynamics of brain tissue [22], [23]. Altered diffusion properties could reflect the variations of fiber density, myelination and cell membrane permeability in the context of physiological and pathological scenarios [24], [25]. In this study, we aimed to explore the diurnal microstructural alterations of healthy human brain using DTI and voxel-based morphometry (VBM), providing a baseline for researches of clinical neuroscience, sleep medicine, psychology and psychiatric disorders.

## Materials and Methods

### MR Data Acquisition

This study was approved by local Institutional Review Board of CAS-SIAT (Chinese Academy of Sciences, Shenzhen Institute of Advanced Techmology). Subjects provided their written consent prior to the participation of the study. Fifteen right-handed healthy participants (6 male and 9 female, 23–31 years, mean age 24.8±2.1 years) were recruited from graduate students who live on campus and undergo consistent routine level of daily activities. Informed consent was obtained from all subjects prior to the MR examination. Medical history was reviewed carefully for each subject to rule out endocrinal, neurological and/or psychiatric illnesses before qualifying for imaging. No participant has current history of drug, coffee, smoking or alcohol abuse. T1 weighted, T2 weighted and FLAIR imaging with routine clinical protocols were performed for the purpose to exclude subjects with white matter lesions.

MR data were obtained on a 3T scanner (Trio system, Siemens Magnetom scanner, Erlangen, Germany) with a 12-channel head coil in a scan room with temperature controlled between 23°C to 24°C. High resolution T1 weighted images were acquired for the entire brain using MPRAGE sequence for anatomical reference with TR/TE/TI = 1900/2.53/900 ms, flip angle = 9°, FOV = 250 mm, slice thickness = 1 mm, acquisition matrix = 256×256. DTI scan was performed with single-shot spin echo echo-planar imaging (EPI) sequence with TR/TE = 4300/104 ms, FOV = 250 mm, image matrix = 128×128, slice thickness = 3 mm, non-zero b value of 1000 s/mm^2^ in 20 gradient directions. Integrated parallel acquisition technique (iPAT) with acceleration factor of 2 was used to reduce the acquisition time and image distortion from susceptibility artifacts.

For each participant the MR scans were performed in the morning (nominated as AM, 8∶30 a.m.±0.50 h) and repeated in the evening (nominated as PM, 7∶30 p.m. ±0.50 h) during a 24-hour interval. The consistency of the DT MRI sequence over time was tested using 5 scans in the evening with a cylindrical MRI phantom filled with a solution of 375 g NiSO_4_ and 5 g NaCl per 1000 g H_2_O distillation using the proposed imaging parameters in this study.

### Data Processing

Image preprocessing was performed using DtiStudio (version 2.4; Johns Hopkins Medical Institute, Laboratory of Brain Anatomical MRI, http://cmrm.med.jhmi.edu/). Distortion induced by eddy currents and simple head motions were corrected by an affine transformation algorithm. Parametric maps of fractional anisotropy (FA), apparent diffusion coefficient (ADC) and eigenvalues (λ_i_, i = 1, 2, 3) [26] were calculated pixel by pixel. FA measures the fraction of the anisotropic diffusion, while ADC demonstrates the magnitude of diffusion of water molecules within tissue, eigenvalue λ_1_ represents the axial diffusivity of water molecules along the principal orientation of tissue structure (λ_//_ =  λ_1_), and radial diffusivity (λ_⊥_ = (λ_2_+λ_3_)/2) represents the water diffusivity in the direction perpendicular to the principal orientation of the cerebral microstructure. Voxel-based analysis was performed with SPM8 software package (Statistical Parametric Mapping; Wellcome Department of Cognitive Neurology, University College London, UK; http://www.fil.ion.ucl.ac.uk/spm) implemented in Matlab (Mathworks, Massachusetts, U.S.A., version 2010b). Two-step normalization was performed as described in previous studies [27], [28]. The first step was to align the images without diffusion weighting (b = 0 s/mm^2^) to the Montreal Neurologic Institute (MNI) space by using the EPI template supplied with SPM8 to estimate the normalization parameters which were then used to transfer the FA, ADC and λ_i_ (i = 1, 2, 3) maps with voxels resampled to 3 mm×3 mm×3 mm. Then, the normalized maps were smoothed using a Gaussian kernel with a full width at half maximum of 6 mm.

### Statistical Analysis

Diurnal variations in FA, ADC, λ_//_ and λ_⊥_ for each voxel across the entire cerebrum were examined using a paired t test with REST toolkit [29] implemented in Matlab. P<0.05 was considered to be statistically significant with a cluster size >168 voxels (AlphaSim corrected). Statistical maps obtained from the above steps were superimposed on the anatomical template Ch2.nii to facilitate visual inspection.

Clusters with significant morning-evening diffusion variations were extracted as regions of interest (ROIs). The percentage of the diurnal variation in DTI indices was calculated by (Index_am_ – Index_pm_)/Index_am_×100% for each of the parameters of FA, ADC, λ_//_ and λ_⊥_ which were averaged within the ROI.

### Repeatability Assessment

The computation of a numerical signal-to-noise ratio (SNR) value for DTI experiments is not straightforward, as diffusion weighted images and b0 images have different signal intensity and noise profiles. Since the SNR in a selected anatomical location in DWIs is dependent on the diffusion direction [30], the repeatability of the SNR between the two scans of this study was typically investigated on b0 images based on the following calculation: 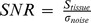, Where 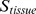 is the average signal of brain tissues in ROIs of genu of the corpus callosum (GCC), corona radiata (CR) and centrum semiovale (CS), 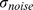 is the mean value of the four standard deviations of the noise in ROIs of the background.

Consistency of the DT MTI sequence was assessed in the calculated FA and ADC maps from the consecutive 5 scans of MRI phantom. A circular ROI at the center of slice 14 with a radius 20 mm was selected for analysis. The FA and ADC values of each pixel inside the ROI were obtained and the mean and standard deviation (SD) of FA and ADC were calculated for each scan. The SD obtained over the five scans was treated as the uncertainty of the measurement. ADC values of the five scans were fitted to a linear regression function to eliminate the effect of radio frequency heating on the measurement. The standard error of the linear fit is considered as the uncertainty of each ADC measurement. The intercept of the first scan was considered as the “mean” ADC value.

## Results

### Interscan Consistency of the DT MRI

**Figure 1:**
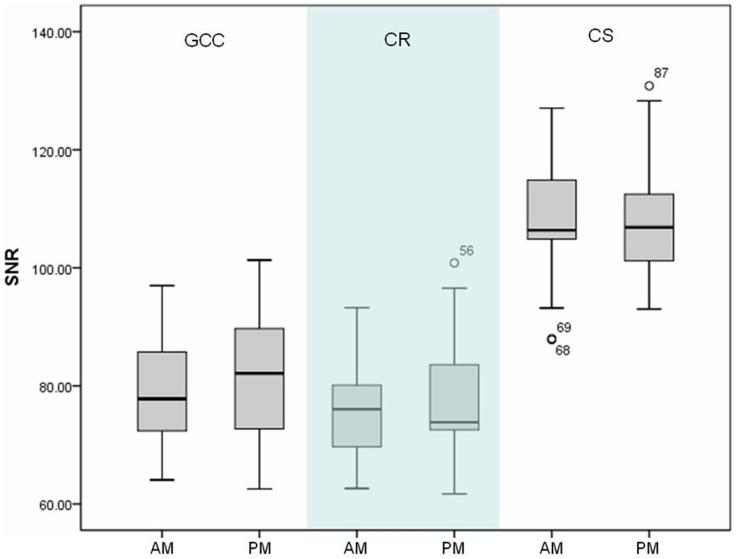
Interscan SNR variation. Box-and-whisker plot of SNR calculated for all subjects as a function of scan sessions for ROIs in genu of corpus callosum (GCC), corona radiata (CR) and centrum semiovale (CS).

The numerical SNR of the repeated scans ranged from 76.7±9.2 to 108.2±10.9 for GCC, CR and CS. No significant inter-scan difference in the SNR was revealed for any of the aforementioned ROIs (p = 0.09, 0.50 and 0.60 for GCC, CR and CS, respectively) (Figure 1).

For the uncertainty assessment, the FA value over five scans was 0.0522±0.0162, and the uncertainties of these FA value were 0.0056±0.0015. The ADC value over five scans was 1.8106±0.0330 (10^−3^ mm^2^/s), and the uncertainties were 0.0015±0.0023 (10^−3^ mm^2^/s). The uncertainties of the measured DTI scans were on the acceptable order of 0.001 for the purpose of the current study.

### Diurnal Variations of the Diffusivity of Brain Water

**Figure 2:**
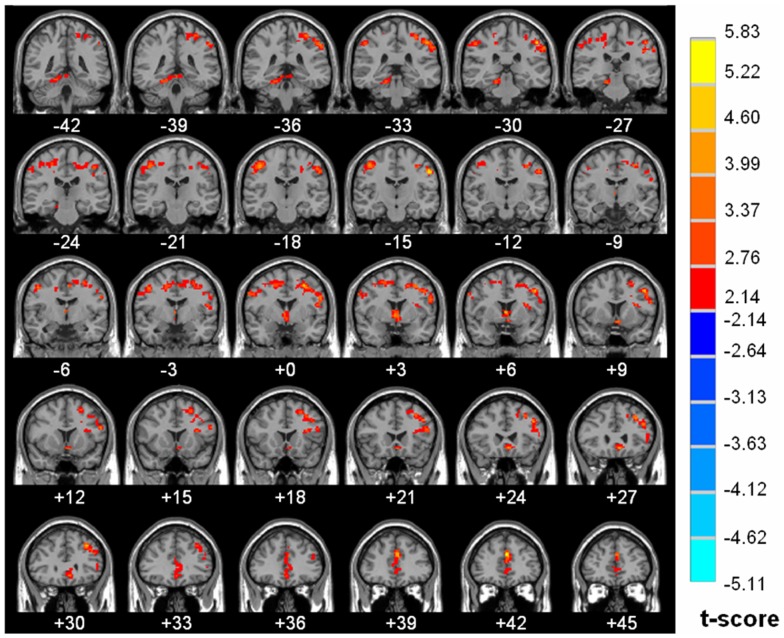
Diurnal variations of FA map. Parametric map superimposed on the Ch2.nii template locates the neuroanatomical regions with significantly increased morning fractional anisotropy (FA) relative to the evening measurement (P<0.05, cluster size >168 voxels). The left hand side of the image is the right side of the brain (radiological representation). Color bar indicates t value scale (Red-yellow, AM >PM, blue-green, AM<PM).

**Figure 3:**
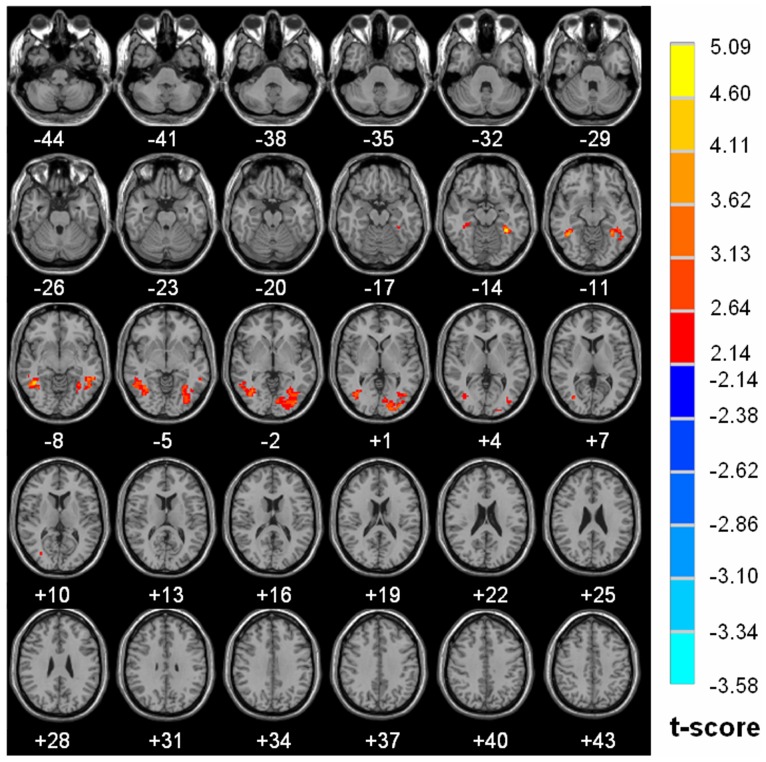
Diurnal variations of ADC map. Axial sections show areas with significantly increased morning ADC (P<0.05, cluster size >168 voxels). The results are mapped on the Ch2.nii template. The left side of the image is the right side of the brain (radiological representation). Color bar indicates t value (Red-yellow, AM >PM, blue-green, AM<PM).

**Figure 4:**
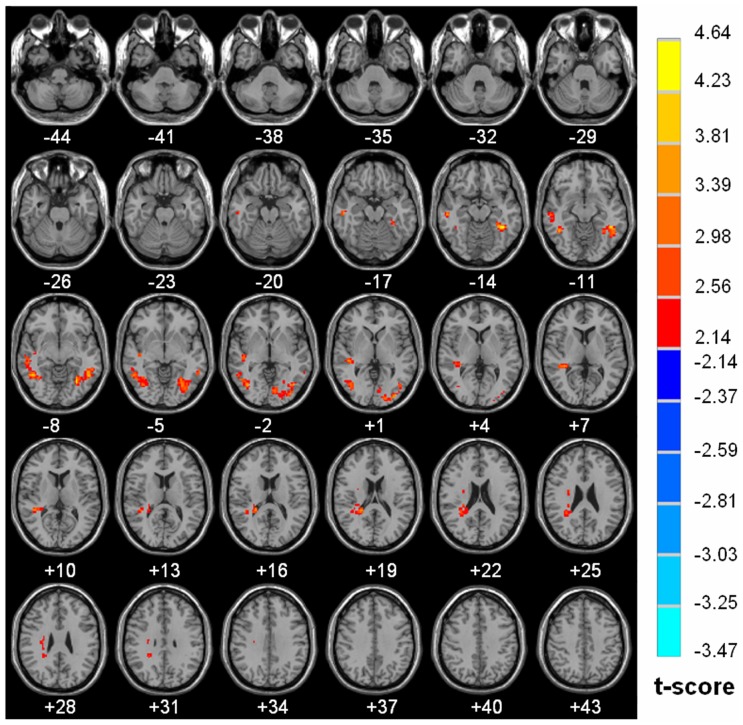
Diurnal variations of λ_//_ map. Brain areas with significant increase of λ_//_ in AM than in PM (P<0.05, cluster size >168 voxels). The results are projected on the Ch2.nii template. The left side of the image is the right side of the brain (radiological representation). Color bar indicates t value (Red-yellow, AM >PM, blue-green, AM<PM).

**Figure 5:**
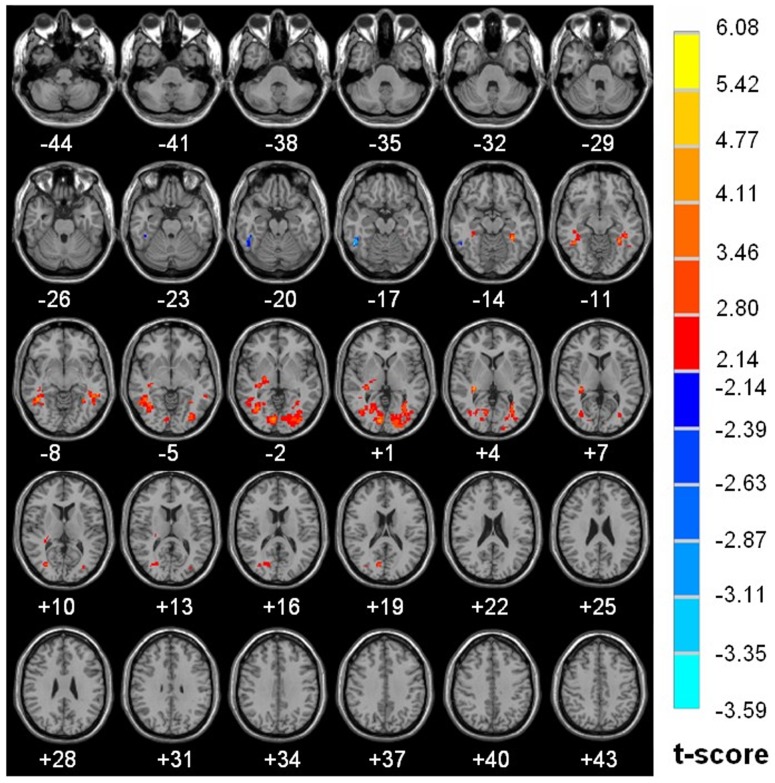
Diurnal variations of λ_⊥_ map. Brain regions with significant diurnal variations of λ_⊥_ between AM and PM (P<0.05, cluster size >168 voxels). The results are projected on the Ch2.nii template. The left side of the image is the right side of the brain (radiological representation). Color bar indicates t value (Red-yellow, AM >PM, blue-green, AM<PM).

The detail of brain regions with significant diurnal changes in water diffusion, cluster volume and peak MNI coordinates were summarized in Table 1. Strong AM to PM contrast in FA (8.02% to 20.50%) was predorminantly seen in both right (20.50%) and left (17.10%) subgyral whiter matter of bilateral frontal and parietal lobes, specifically located in precentral gyrus Brodmann area 4 (BA4), middle frontal gyrus (BA 9), postcentral gyrus (BA 3, BA 2), premotor and supplementary motor cortex (BA 6), precuneus (BA 7), Wernicke’s area (BA 40), anterior cingulated (BA 32), caudate, thalamus (MNI coordinate 3 −6 9 and −3 −5 9) (Figure 2). Significantly increased morning measurements on the right and left hemisphere were observed for ADC (R 4.43% vs. L 5.60%), λ_//_ (R 4.33% vs. L 5.84%) and λ_⊥_ (R 4.03% vs. L 5.57%) in regions of bilateral occipital and temporal lobes including middle occipital, inferior occipital, lingual, dorsal and posterior fusiform (BA37), middle temporal and inferior temporal gyri, as shown in Figures. 3, 4 and 5 respectively. There is no hemispheric difference in the change rate of the diurnal variations of brain water diffusivity for ADC (p = 0.77), λ_//_ (p = 0.08) and FA (p = 0.25). Diurnal variation of λ_⊥_ was significantly left side dominant (5.57% vs. 4.03%, p<0.05). The average change rate of ADC, λ_//_, λ_⊥_, and FA in brain areas without significant AM-PM contrast were 1.59%, 2.15%, 1.20% and 2.84%, respectively.

**Table 1:** Summary of neuroanatomical regions with significant diurnal variations in FA, ADC, λ_//_ and λ_⊥_.

Indices	Brain region	Peak coordinate	Cluster seize	AM value	PM value	(AM-PM)/AM %
FA	Precentral_L	(−54 −15 42)	1154	0.13±0.02	0.11±0.02	17.10
	Postcentral_L					
	Parietal_Inf_L					
	Precuneus_L					
	Frontal_Sup_L					
	Precentral_R	(45 −18 51)	446	0.14±0.04	0.11±0.02	20.50
	Postcentral_R					
	Precuneus_R					
	Parietal_Inf_R					
	Frontal_Sup_R					
	Cingulum_Ant_L	(0 42 24)	192	0.16±0.02	0.14±0.01	9.90
	Frontal_Sup_Medial_L					
	Frontal_Med_orb_L					
	Caudate_L					
	Thalamus_L					
	Cingulum_Ant_R	(0 42 24)	95	0.15±0.01	0.14±0.01	8.02
	Frontal_Med_orb_R					
	Frontal_Sup_Medial_R					
	Caudate_R					
	Thalamus_R					
ADC(*10^−4^ mm^2^/s)	Sub-gyral	(−33 −39 −15)	284	8.55±0.48	8.07±0.48	5.60
	Occipital_Mid_L					
	Occipital_Inf_L					
	Fusiform_L					
	Lingual_L					
	Temporal_Mid_L					
	Sub_gyral	(45 −48 −9)	168	8.36±0.48	7.99±0.55	4.43
	Temporal_Inf_R					
	Temporal_Mid_R					
	Fusiform_R					
	Middle Occipital Gytus					
λ_//_(*10^−4^ mm^2^/s)	Sub-gyral	(−42 −42 −15)	255	10.24±0.51	9.64±0.56	5.84
	Temporal_Inf_L					
	Occipital_Inf_L					
	Fusiform_L					
	Occipital_Mid_L					
	Lingual_L					
	Fusiform Gyrus					
	Temporal_Mid_L					
	Sub-gyral	(45 −51 −9)	185	10.03±0.42	9.6±0.54	4.33
	Temporal_Inf_R					
	Temporal_Mid_R					
	Middle Occipital Gyrus					
	Fusiform_R					
	Occipital_Inf_R					
λ_⊥_ (*10^−4^ mm^2^/s)	Sub-gyral	(9 −84 0)	407	7.36±0.47	7.07±0.47	4.03
	Occipital_Mid_L					
	Occipital_Inf_L					
	Lingual_L					
	Fusiform_L					
	Temporal_Inf_L					
	Temporal_Mid_L					
	Cuneus					
	Sub-gyral	(−33 −39 −15)				
	Temporal_Inf_R					
	Lingual_R					
	Calcarine_R					
	Cuneus					
	Fusiform Gyrus					
	Occipital_Mid_R					
	Temporal_Mid_R					
	Temporal_Inf_R (aal)	(54 −48 −18)	27	5.4±1.9	6.4±1.9	−18.58
	Fusiform Gyrus					

The AM value and PM value are given as intersubject mean ± intersubject standard deviation.

Prominent PM to AM variation (18.58%) was detected in the grey matter of right inferior temporal and fusiform gyri (BA37) for λ_⊥_ (Figure 5).

## Discussion

In this preliminary study, we identified significant diurnal variations of cerebral microstructure indexed by FA, ADC, λ// and λ_⊥_ of healthy adult brain.

### Diurnal Alterations of Water Diffusion Properties in Brain

ADC has been proved to be sensitive to the changes of tissue microstructure [31], [32]. Transient decreased ADC was detected in the visual cortex during neuronal activation, which was ascribed to the increased restriction of water diffusion and extracellular tortuosity due to cell swelling following the evoked potential [33], [34].

Significant morning to evening contrast of ADC in this study was considered a reflection of the diurnal microstructural changes of human brain. However, effects from vascular sources and body temperature may partially contribute to the diffusivity variation. Blood flow variation induced by hypercapnia was reported to contribute up to 2% signal changes in DWI with b values of 1000 s/mm^2^ to 1200 s/mm^2^ in the absence of changes in neuronal firing [35], and ADC varies 1.83% to 1.89% when temperature rises from 36°C to 38°C [36]. Given the fact that blood flow was lower in the morning and the core body temperature fluctuates less than 1°C daily with the nadir at about 3 hours before wakening and at 90-degrees out of phase with the cerebral blood flow velocity [37] for human being, the diurnal ADC variations in this study was less likely resulted from the composite effect of vasculature and temperature at the time of data acquisition. Instead, the endogenous circadian machinery conserved from evolution was assumed to play the pivotal role in mediating the diurnal changes [38].

It’s been well established that superochiasmatic nuclei (SCN) retaining the circadian rhythm of gene expression and electrophysiological activity [39], receive photic input directly from the retina and synchronize to the light-dark cycle [40]. Retina bears an inbuilt circadian mechanism that regulates the light-dark adaptation of phototransduction in the visual pathway [41]. These endogenous biorhythms may contribute to the vital changes in the neural activities at the levels of cell, tissue and organ along the visual pathway, which in turn results in measurable microstructural alterations reflected by water diffusivity, as observed in the visual cortex in the current study.

The AM - PM change rate of the radial and axial diffusivity were in the similar trend and scale as ADC, which were assumed to comply with the same endogenous rhythmic mechanism in addition to their mathematical relationship (ADC = *λ*_//_/3+2/3 λ_⊥_). Changes in *λ*_//_ and λ_⊥_ were reported to be closely related to the alteration of myelination and axonal density, respectively [42]. The increased morning λ_⊥_ in the middle occipital, inferior occipital, lingual, fusiform, middle temporal and inferior temporal gyri in this study may reflect the overall morphological effect including changes of axonal density or caliber and the interaction between the intra- and extracellular compartments in response to the varied level of neural firing, suggesting that these brain areas are more integrated architecturally and less tortuous in the extracellular space in the morning relative to the evening. However, cautions should be taken in the data interpretation involving *λ*_//_ and λ_⊥_, as they do not represent independent components of water diffusivity in DTI. Changes of *λ*_//_ can cause a fictitious change in λ_⊥_ and vice versa in voxels with crossing fibers [43].

FA, as the most frequently employed index, is closely associated with the microstructural features of cerebral white matter [44], [45]. Changes in FA indicates the altered directional organization and architectural integrity of cerebral white matter [18], [46] that may elucidate the physiological underpinnings following a task related neural activity [47]. The increased morning FA in the bilateral frontal and parietal lobes in this study may imply the diurnal variations in the structural plasticity and functional recruitment of astrocyte-neuron interaction in response to the daytime enhanced neural activities in these regions [48]. Specifically, functions of motor skill, reasoning, alertness, high level cognition and expressive language are mainly coordinated in these areas in which neural activities are more intensively performed in the daytime relative to the evening, as increased FA indicates improved functional performance [49]. In addition, other mechanisms may yield remarkable diurnal morphological changes in these brain tissues, as observed in the glial cells in SCN [50]. However, this hypothesis and its underlying mechanisms remain to be clarified.

Substantial PM to AM contrast of λ_⊥_ was seen in the ventral fusiform gyrus of the right hemisphere (BA37) only. As part of the human visual system, the ventral fusiform gyrus contains structures specialized for facial recognition [51] and fine discrimination between categorical objects with right hemispheric lateralization [52]. Visually evoked activities in early visual areas (BA17, BA18) may reflect a nonconscious memory of face and different object categories, while in late visual regions (BA19, BA37) it was believed to involve a greater activity associated with the conscious memory of previous hits [53]. Experiment on face recognition memory showed a higher hit rate and false alarm rate in subjects who started the test at evening (9 pm) than those who underwent the experiment during the daytime [54]. The significant PM-AM λ_⊥_ contrast in BA37 in this study may function as the substrate of the enhanced neural activity in the ventral fusiform for the consolidating process of memory for face recognition and categorical object discrimination at evening.

As part of the central control of biorhythm, thalamus manifests diurnal phases in correspondence to gene expression and hormonal releasing [55]. In this study, morning-evening contrast was detected in a cluster as small as 8 voxels in thalamus, which corresponds to the anatomical and physiological territory of the right and left medial dorsal nucleus of the thalamus (MNI coordinate (3 −6 9) and (−3 −5 9), respectively). However, cautions should be taken in the interpretation of the data, as the effect from noise or other sources of contamination cannot be ruled out.

Sleep deprivation and insomnia pose detrimental effect on the function and microstructure of the brain [56], [57]. The regional morning-evening contrast of the microstructural changes in this study were assumed intrinsic to the circadian rhythm in which natural sleep is thought to reflect the dynamics of the rhythmic properties instead of being causative to the microstructural changes over night [58].

### Hemispheric Specialization of the Diurnal Changes of Water Diffusion in Brain

Functional and structural laterality are fundamental properties of human brain [59]–[61]. Neuroimaging data has revealed the structural hemispheric asymmetry in cerebral white matter [62], [63]. Previous PET studies demonstrated that functionally, a right lateralized intrinsic network including frontal and parietal lobes for alertness and attention is more intensive in the morning [64], [65]. However, microstructurally, a greater diurnal variation of FA was observed in the right frontal and parietal lobes but did not reach the statistical significance in the current microstructural study. The PM-AM variation of λ_⊥_ showed a significant left side predominance in this study in regions of middle occipital, inferior occipital, lingual, fusiform (BA 37), middle temporal and inferior temporal gyri which are part of the higher levels of visual processing for complex object features identification, distance contemplation and visual memory encoding. This finding was in agreement with a previous research that perception of local features was lateralized to the left occipital lobes in which the asymmetry was proposed to originate from the differential frequency bias between the hemispheres [66], [67] or from the differential encoding beyond the sensory level [68].

### Limitations of Current Study

There are several limitations in this study. The comparison of SNR between the morning and evening MR examinations was based on a ROI-wise analysis. Parallel imaging technique with phased-array coil was used during the data acquisition, which could produce an inhomogeneous SNR profile and the conventional method for noise quantification is not recommended. The simplified SNR comparison between the two scan sessions with the same setup for each subject in this study is expected to minimize instead of annihilating the noise input to the diurnal variations of the water diffusivity measured by DTI, making it reasonable to attribute the diurnal variation of water diffusivity to the intrinsic properties of human brain.

Cardiac pulsation can be another source inducing significant variability in estimates of diffusion tensor quantities [69]. Considering the acquisition time and the efficiency of cardiac gating, we didn’t use the cardiac gating protocol in this study. Habib and colleagues demonstrated that non-gated DTI tends to overestimate the FA and mean diffusivity in some individuals, but the cardiac pulsation effect was within a negligible scale in group studies [70]. This may alleviate, although not completely eliminate the concern of the cardiac pulsation effect on the calculation of the diffusion tensor quantities for the current study.

Only morning and evening data of young subjects were examined in this study. Small sample size may have limited the statistic power and made the interpretation of the findings more challenging. In addition, behavioral features were not checked in the context of the diurnal microstructural changes. It would be more informative for future studies to investigate the circadian features of the brain in a larger cohort.

## Conclusions

Significant diurnal microstructural variations were observed in healthy adult human brain indexed by FA, ADC, axial and radial diffusivities, which may reflect the intrinsic properties of neural rhythm of human brain in correspondence to the environment adaptation in a light-dark cycle. This research provided a helpful baseline toward promising translational applications in neuroscience, sleep medicine, psychological and psychiatric disorders, and necessitates that diurnal effect should be taken into account in following up studies using diffusion tensor quantities.
